# Supplementary material for: Gendered perspectives of yoga in the Key Stage 1 classroom: Qualitative content analysis indicates contrasting views of teachers and pupils
Source: PLoS One. 2026 Feb 24;21(2):e0343622. doi: 10.1371/journal.pone.0343622 (PMC12931780; doi:10.1371/journal.pone.0343622)
Supplement: S1 File — This document provides details of the Qualitative Content Analysis (QCA) procedure used in this study, as well as the full QCA coding frame for the teachers’ and pupils’ data. (PDF) [file pone.0343622.s001.pdf]

Qualitative Content Analysis (QCA)

The authors followed the eight steps of QCA as described by Schreier, 2014 [1] as described below.

Step 1. Deciding the research question(s)
This study aims to provide information towards the following two research questions: Q1) From teachers' perspectives, how do male and female Key Stage 1 (KS1) pupils engage with school-based yoga? Q2) What are the attitudes and opinions of male and female KS1 pupils towards these activities?

As described in the manuscript, the researchers' original intent was not specifically to explore the gendering of yoga, but as this topic emerged spontaneously during teacher interviews, these research questions were devised after data collection.

Step 2. Selecting material
Recordings of the teacher interviews, and the pupil qualitative tasks, were transcribed in full, verbatim, including the researcher's questions and comments, with any personally identifying details removed at this stage. Upon reading and re-reading the transcriptions, all material that seemed relevant and interesting regarding the research questions were selected [2] with care taken to ensure that the selected material reflected the full diversity of participants [3]. To this end, selected material contained all relevant quotes, including those that differed from or contradicted the views expressed by other participating teachers / pupils. For instance, the children provided a range of responses when asked to describe whether yoga is 'for girls / for boys / for everyone', and all responses were selected, regardless of consistency with traditional gendered views of sport / athleticism / physicality.

Step 3. Building a coding frame
This stage is theory-driven (i.e. deductive) and for the present study involved literature relating to Gender Schema Theory (GST) and the gendering of yoga. From this literature review, the authors summarised that, in general, yoga is considered a feminine / female activity by adults [4] and adolescents [5] but it is not yet known whether young schoolchildren and their teachers hold similar gendered views. As such, the main category in the coding frame was defined as, 'Information relating to teachers' and pupils' gendered views of yoga', alongside the following two subcategories:
i) Views regarding what yoga entails, and who is more suited to, more engaged with, or better at, yoga
ii) Pupils' apparent confidence and comfort when demonstrating an interest in, aptitude for, or knowledge of yoga'.

Step 4. Segmentation

During this step, the selected material is divided into segments (called 'coding units'), with each segment representing a single subcategory, while retaining enough context to be meaningful [6]. Thus, individual coding units were identified which comprise those parts of the material that could be interpreted in a meaningful way according to the two subcategories described in the coding frame.

The full coding frame – which contains all relevant data from teachers and pupils – is provided below. As advised by Schreier (2014) [7], each code has been given a name / title and a description of its features, as well as examples from the data where the code was identified, alongside decision rules to clarify when a code is and is not to be used. Additionally, frequency counts are included to show how many participants made comments relating to each code.

Step 5. Trial coding
Categories, subcategories, and codes are then applied to all selected material. As such, we used the full coding frame to categorise the selected teachers' and pupils' data. During this stage, the wording of individual codes was adjusted and amended to best fit the data.

Step 6. Evaluating and modifying the coding frame
This stage involves the evaluation of whether the categories, subcategories, and codes in the coding frame adequately describe the material and the concepts underlying the research questions. If not, changes and / or additions are required. For example, any overlap between (sub)categories or uncertainty with regards to when a (sub)category is and is not to be used requires further definition within the coding frame with clarified decision rules if necessary. Once this step was complete, we had finalised the details of one main category, two subcategories, and seven codes which could be used to categorise and summarise all selected material.

Step 7. Main analysis
All selected material is coded during this stage, with the results prepared so that they may suitably answer the research questions.

Step 8. Presenting and interpreting the findings
Finally, all authors reviewed and discussed the findings and conclusions, before writing the manuscript for dissemination. N.B. When drafting and finalising this paper, we used Elo et al.'s (2014) QCA checklist [8] to support the validity of our reporting.

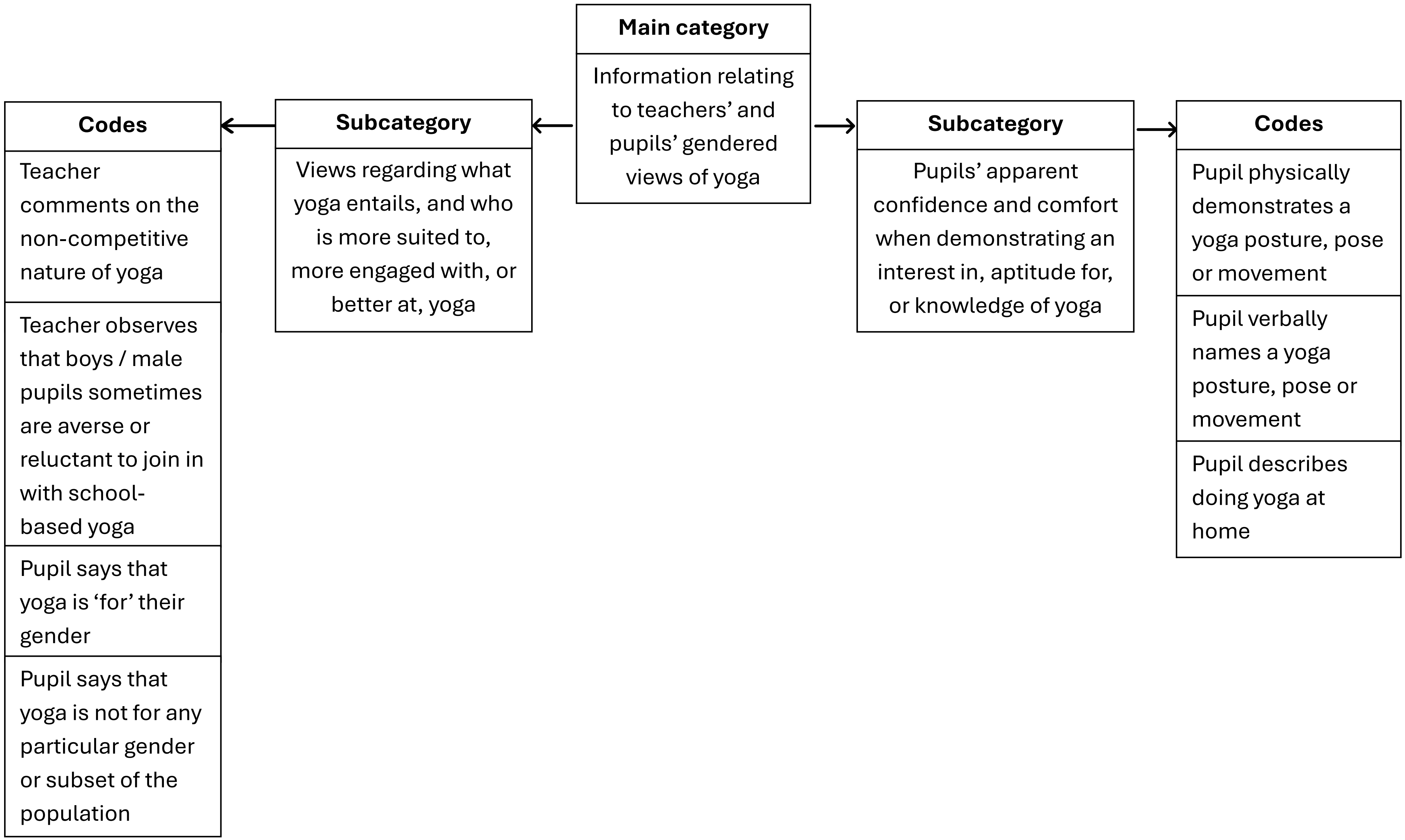
Main category
Information relating to teachers' and pupils' gendered views of yoga
Subcategory
Views regarding what yoga entails, and who is more suited to, more engaged with, or better at, yoga
Codes
Teacher comments on the non-competitive nature of yoga
Teacher observes that boys / male pupils sometimes are averse or reluctant to join in with school-based yoga
Pupil says that yoga is 'for' their gender
Pupil says that yoga is not for any particular gender or subset of the population
Subcategory
Pupils' apparent confidence and comfort when demonstrating an interest in, aptitude for, or knowledge of yoga
Codes
Pupil physically demonstrates a yoga posture, pose or movement
Pupil verbally names a yoga posture, pose or movement
Pupil describes doing yoga at home

Main category	Subcategory	Code	Features	When to use	When not to use	Examples / Quotes
Information relating to teachers' and pupils' gendered views of yoga	Pupils' apparent confidence and comfort when demonstrating an interest in, aptitude for, or knowledge of yoga	Physically demonstrates yoga pose, posture or movement<br><br>19 pupils<br>10 girls<br>9 boys	Child moves or positions her/himself in such a way as to physically demonstrate a yoga movement, pose, or posture	When a child makes any physical demonstration of their yoga learning, form brief 'prayer hands' to more complex movements	When a child makes a movement that is not recognisable as a yoga pose / posture, and gives no verbal description of their movements being related to yoga	MP4 "when you sit down and do that "[hands in prayer at chest].<br><br>MP5 [hands in prayer at chest with eyes closed]<br><br>MP4: [hands in prayer at chest with eyes closed]<br><br>MP6: [hands in prayer at chest] TWICE<br><br>MP6: [sits cross-legged, hands in Gyan mudra - thumb touching middle finger, then stands and puts hands in prayer at chest]<br><br>MP4: [hands in prayer at chest with eyes closed]<br><br>MP4: [hands in prayer at chest]<br><br>MP2: [sits cross-legged with hands in prayer at chest]<br><br>MP6: stands with hands in prayer at chest then

						sits cross-legged, hands in prayer, closes eyes and bows head]<br><br>FP7 [tree pose]<br><br>FP6 [tree pose]<br><br>FP3 [energetic kick]<br><br>FP1 [hands in prayer at chest]<br><br>FP6 [dancer pose]<br><br>FP7 [hands in prayer at chest]<br><br>FP6 [tree pose]<br><br>FP7 [tree pose]<br><br>FP7 [dancer pose]<br><br>FP6 [tree pose – but with arms at side of body]<br><br>FP5 [tree pose]<br><br>FP7 [straightening from bent knees while raising arms up and to the sides]

						MP8 [prayer hands, one leg balance with other foot on inside knee – tree pose]<br><br>MP7 [Cobra pose]<br><br>MP8 [prayer hands, one leg balance with other foot on inside knee – tree pose]<br><br>MP11 [prayer hands, one leg balance with other foot on inside knee – tree pose]<br><br>MP7 [prayer hands, one leg balance with other foot on inside knee – tree pose]<br><br>MP10 [Downward dog]<br><br>MP9 [prayer hands, one leg balance with other foot on inside knee, head bows]<br><br>MP9 [Downward dog]

						MP7 [side-angle pose, one arm straight up – triangle pose]<br><br>MP8 [prayer hands, one leg balance with other foot on inside knee – tree pose]<br><br>MP11 [Downward dog]<br><br>MP8 [open mouth, tongue stretched out down to chin]<br><br>MP7 [eyes wide, open mouth, tongue stretched out down to chin]<br><br>MP9 [sits cross-legged, hands resting on knees, eyes closed, head bowed]<br><br>MP9 [hands in prayer, head bowed]<br><br>FP8 [cobra]<br><br>FP8 [extended cobra upwards]<br><br>FP8 [beginning part of sun salutation]

						FP10 [cobra – with toes touching head]<br><br>FP11 [stand in tree pose “flamingo” with arms outstretched, balance on one foot with the other resting on the inside of the other ankle]<br><br>FP12 [stand in tree pose “flamingo” with arms outstretched, balance on one foot with the other resting on the inside of the other ankle]<br><br>FP9 [stand in tree pose “flamingo” with arms outstretched, balance on one foot with the other resting on the inside of the other ankle]
		Verbally names a yoga pose, posture or movement<br><br>11 pupils<br>5 girls<br>6 boys	Child provides a name or title for a yoga movement or pose, including non-formal child-friendly terms	When a child verbally names a yoga movement or pose	When a child describes another feature of their yoga experience in school (e.g., the name of a yoga instructor from a YouTube video)	FP6 “tree”<br><br>FP5 “tree pose”<br><br>FP4 “we do the dog”<br><br>MP10 “cobra”<br><br>MP11 “cobra”

						MP7 “cobra”<br><br>MP10 “I’m, I’m the downward dog”<br><br>MP8 “Downward dog”<br><br>FP10 “cobra”<br><br>FP8 “we went up like a seal”
		Describes also doing yoga at home<br><br>7 pupils<br>3 girls<br>4 boys	Child describes also practicing yoga at home, either alone or with other family members	When a child mentions taking part in yoga activities at home, either currently or in the past	When a child describes that one of their family members does yoga, but does not say that they join in / take part with them	MP1 “I do it sometimes, and like we do – ” [inaudible]<br>*KW: You do it with your mam*<br><br>FP7 “In the house… I do it myself”<br><br>MP10 “I, I do yoga at my house”<br><br>MP11 “I do it both. School and my house”<br><br>MP9 “I usually do it ev, every Saturday. I just do it at home in my special compartment. I have a video that I watch”

						MP10 "I do too, I have a video too"<br><br>MP9 "and I, and I do it in the den under the stairs. It has music and a story about breathing, and I practice it quite a lot"<br><br>FP10 "sometimes I do it at home"<br><br>FP8 "My sister does, used to do yoga in the summer holidays with me"
	Views regarding what yoga entails, and who is more suited to, engaged with, or better at, yoga	Teacher recognises the non-competitive nature of yoga<br><br>5 teachers	When a teacher comments on the non-competitive nature of yoga	A teacher denies that they have observed any competition between pupils during yoga activities	When a teacher speaks about competition in general. When this occurs, but if there is no reference to gender or yoga, it is not selected for coding	*"I think it was very much, each like in their own individual space. Because they weren't working together with a partner or as a, like a team, I don't think they were ever really aware of that, that, er, that, there were other people next to them that they could compare themselves with necessarily"* (Ppt 3)<br><br>*"It* [competition] *is not something I've consciously noticed...no, I've not. That's not*

						something I would say I have witnessed personally" (Ppt 4)<br><br>*"There's never been kind of a competitive streak, which is really when you say it like that you think, oh, actually because if they're out in the field doing their sports day, or their races, straight away it's there, but actually no, it's never really shown… They know that it's the team effort. It's for us, and I think that's probably why it kind of it doesn't have that competitive element maybe"* (Ppt 6)
		Teacher observes that boys / male pupils sometimes are averse or reluctant to join in with school-based yoga<br><br>5 teachers	A teacher gives examples of male pupils that are reluctant to engage with yoga, or describes a general observation that boys seem somewhat averse to these activities	When a teacher speaks about male pupils' engagement with yoga activities	When a teacher speaks about how their class as a whole typically engages with yoga. When this occurs, without any reference to gender, it is not selected for coding	*"I mean some of the boys weren't brilliant at first with joining in"* (Ppt 2)<br><br>*"Little boys, like boys. I, and I think that then it would become more sort of hyped up… just find it a little bit too much, you know"* (Ppt 4)<br><br>"*Like one of the children* [a male pupil], *wasn't*

						really engaging particularly well with it…I don't think they felt comfortable doing it…I think it was just he didn't want to do it in front of other people, and didn't like doing it. I think he saw it a bit like a dance, that sort of thing, and just didn't want to do it" (Ppt 5)<br><br>*"The ones that I would say maybe don't find – they give it a go, but they're not as engaged as the other ones, erm, it's some of the boys".* (Ppt 6)
		Male / Female pupils say that yoga is 'for' their gender<br><br>10 pupils<br>5 girls<br>5 boys	A male / female pupil states that yoga is 'for' their gender, or states that their gender is better at, or more capable of yoga than the other	When a male / female pupil makes any statement (prompted or spontaneous) that yoga is 'for' their gender, or that their gender is better at, or more capable or, or more suitable for yoga than the other	When a child physically demonstrates how well they can hold a certain pose. In this case, the subcategory 'Physically demonstrates pose' above is appropriate	MP3 "It's, it's boys"<br><br>MP1 "Only for boys"<br><br>KW are boys better at yoga than girls?<br>MP10 "Yeah"<br><br>MP8 "Yes"<br><br>MP9 "Yeah we're better. Boys… Boy, boys usually have better balance and manage to stay in poses

						longer than girls. Because girls aren't usually as strong as boys, so it, it's a bit, well, out of [nods], yeah"<br><br>FP12 "Girls"<br><br>FP10 "Girls"<br><br>FP11 "Girls, because girls are, are more flexible than boys"<br><br>FP10 "Boys, I don't think boys can do like a, like, cobra, you can, erm, cobra, like put their, your legs to their head"<br><br>FP9 "Erm, I don't think boys are very good because I don't think boys do, er, lots of clubs"<br><br>FP8 "girls like take more like practice"

		Pupils say that yoga is not for any particular gender or subset of the population<br><br>9 pupils<br>4 girls<br>5 boys	Child identifies no gender as more suited to yoga	When a child states that boys and girls / males and females are equally suited or equally good at yoga, or states that yoga is for 'everyone'	When a child comments that they personally are good at yoga. When this occurs, without any further reference to gender, it is not selected for coding	FP2: *"Girls and boys"*<br><br>FP7: *"Same"*<br><br>MP5: *"Both – it's boys and girls"*<br><br>"MP9: Any person" [both arms outstretched]<br><br>MP7: *"they're both the same…everyone"*<br><br>MP11: *"Yeah, they're both the same. Both are better…Yeah. Everyone in the world"* [both arms outstretched]

References

[1] Schreier M. Qualitative content analysis in practice. In Flick U (editor) The SAGE handbook of qualitative data analysis. London: Sage Publications, pp. 170–183. Available from: https://www.ufs.ac.za/docs/librariesprovider68/resources/methodology/uwe_flick_(ed-)-_the_sage_handbook_of_qualitative(z-lib-org)-(1).pdf?sfvrsn=db96820_2

[2] Krippendorff K. Content analysis: An introduction to its methodology. Sage publications; 2018 May 9. doi: 10.4135/9781071878781

[3] Schreier M. Qualitative content analysis in practice. In Flick U (editor) The SAGE handbook of qualitative data analysis. London: Sage Publications, page 175 Available from: https://www.ufs.ac.za/docs/librariesprovider68/resources/methodology/uwe_flick_(ed-)-_the_sage_handbook_of_qualitative(z-lib-org)-(1).pdf?sfvrsn=db96820_2

[4] Motzkus CJ, Jarry JL. “Yoga is for girls”: Conformity to masculine norms interferes with yoga engagement in men. Psychol Men Masculinity. 2024 Mar 21.

[5] Uebelacker LA, Wolff JC, Guo J, Feltus S, Caviness CM, Tremont G, Conte K, Rosen RK, Yen S. Teens’ perspectives on yoga as a treatment for stress and depression. Complement Ther Med. 2021 Jun 1;59:102723. doi: 10.1016/j.ctim.2021.102723

[6] Clausen M, Chur-Hansen A, Crabb S, Vincent N. What does flexible work for men really mean?: a qualitative content analysis of key stakeholder accounts. Labour Ind. 2024 Jul 2;34(3):339-59. doi: 10.1080/10301763.2024.2420297

[7] Schreier M. Qualitative content analysis in practice. In Flick U (editor) The SAGE handbook of qualitative data analysis. London: Sage Publications, pp. 170–183. Available from: https://www.ufs.ac.za/docs/librariesprovider68/resources/methodology/uwe_flick_(ed-)-_the_sage_handbook_of_qualitative(z-lib-org)-(1).pdf?sfvrsn=db96820_2

[8] Elo S, Kääriäinen M, Kanste O, Pölkki T, Utriainen K, Kyngäs H. Qualitative content analysis: A focus on trustworthiness. SAGE open. 2014 Feb 5;4(1):2158244014522633. doi: 10.1177/2158244014522633
