# Supplementary material for: Gendered perspectives of yoga in the Key Stage 1 classroom: Qualitative content analysis indicates contrasting views of teachers and pupils
Source: PLoS One. 2026 Feb 24;21(2):e0343622. doi: 10.1371/journal.pone.0343622 (PMC12931780; doi:10.1371/journal.pone.0343622)
Supplement: Attachment — Submitted filename: 20251020_Review_PLOS-One.docx [file pone.0343622.s002.docx]

Review of PLOS One article

**Introduction**

Really well written introduction section. Some suggestions below:

Because the research is UK based, it could be helpful to link to some UK stats on yoga participation: Cartwright, T., Mason, H., Porter, A., & Pilkington, K. (2020). Yoga practice in the UK: A cross-sectional survey of motivation, health benefits and behaviours. *BMJ open*, *10*(1), e031848.

Line 108-109 – when saying ‘much evidence’ can you link the reader to review level evidence rather than a single study? If no review level evidence is present, then is there more than one study you can refer to, otherwise use more cautious wording.

**Methods**

Really excellent to see and I commend the authors section on positionality. My only critique would be that it would have been helpful to have utilised a ‘critical friend’ from outside of the research team for objectivity – although I know that this can be difficult to access with limited researcher capability/capacity! Maybe something to consider for future qualitative research.

Tables 1-2 – is it necessary to have the locations of the schools/teachers? If not necessary, I would remove to protect anonymity. You could classify the schools with number IDs and add ‘primary/infant’ etc.

Line 188-189 – Some additional detail on the assent processes would be helpful with the children being so young, how did you know they understood what was being asked of them?

Line 191 – How would you define contribution and ‘engaged’ in this setting? Did you have any tactics to involve those who may not have felt as comfortable engaging in the more traditional sense?

Can you add some detail on justification of sample size for your study?

Line 266 – not sure this reference is in the right place?

**Findings**

A really thoughtful section and very interesting to read the contrasting opinions/perceptions between students and teachers.

Line 271-273 – Very hard to understand the meaning of this without knowing in-depth the method used – the figure helps slightly but I wonder if there is another way to describe this. And quite interesting that the analysis of the pupils and teachers has been combined – can you detail why. Was it to avoid duplication or did the data complement each other?

As you are discussing the findings with existing literature in the findings section, should you rename it ‘findings & discussion’?

Line 321-326 – Suggestion: because you are repeating a lot of the quotations to discuss, would there be a better way to format this? To avoid duplication could you present a quote and discuss, instead of presenting all quotes, then discussing them again?

Line 344 onwards – agree with this and would be interesting to discuss how yoga is marketed in this age group compared to other lifespan groups. For example, Cosmic kids is considered quite gender neutral and geared towards younger kids versus Studio You which is powered by This Girl Can.

**Conclusion**

This is written quite differently from typical journal articles and seems slightly repetitive with duplication of what we have just read including the references, rather than summarising the main takeaways. Is there a way to condense this section and make it slightly more punchy – the ‘so what?’ of the research needs to come out a bit more.

Limitations

Good section – only suggestion would be that you could perhaps explore the heterogeneity of yoga and the difficulties of research because of this.
