# Supplementary material for: Gendered perspectives of yoga in the Key Stage 1 classroom: Qualitative content analysis indicates contrasting views of teachers and pupils
Source: PLoS One. 2026 Feb 24;21(2):e0343622. doi: 10.1371/journal.pone.0343622 (PMC12931780; doi:10.1371/journal.pone.0343622)
Supplement: Attachment — Submitted filename: Peer Review of Manuscript PONE.docx [file pone.0343622.s003.docx]

**Peer Review of Manuscript PONE-D-25-54686**

**Title**: *‘He saw it a bit like a dance and just didn’t want to do it’: Qualitative content analysis of Key Stage 1 teachers’ and pupils’ gendered perspectives of yoga*

**Recommendation**: **Major revision**

**1. General assessment**

This manuscript presents a timely and relevant qualitative study examining Key Stage 1 teachers’ and pupils’ gendered perceptions of yoga in the North-East of England. The topic contributes meaningfully to the fields of gender studies, educational psychology, and school-based wellbeing interventions.

The research is grounded in GST and utilises qualitative content analysis to investigate the gendering of yoga in early education. The study is methodologically sound in many respects, adheres to ethical standards, and presents original findings not previously published elsewhere.

However, the manuscript would benefit from several important revisions to strengthen clarity, transparency of analysis, and alignment between claims and evidence. Specific attention is needed in the areas of theoretical elaboration, analytic transparency, and framing of conclusions.

**2. Title and framing**

**Title Revision Required**: The current title foregrounds the teacher’s view ("He saw it a bit like a dance...") and does not adequately reflect the dual focus of the study on both teachers and pupils. It risks misrepresenting the scope, which includes children’s perspectives. A more neutral and inclusive title is advised, ideally reflecting the mismatch between teacher and pupil perspectives.

**3. Clarity and Transparency of Methods**

**Missing content analysis table**

The inclusion of a table summarising QCA categories and subcategories is essential. While a graphical coding frame (Figure 1) is referenced and supplementary materials are noted, the main manuscript would greatly benefit from a table displaying key categories,codes, subcodes, and example quotes, as per best practices in qualitative research reporting.

**Interview protocol**

Although semi-structured interviews are described, the article would be enhanced by including example interview questions, particularly those that elicited gender-related insights.

**Analytic elaboration needed**

The explanation of the QCA process (p. 11,12) could be expanded. More detail on how consensus was reached, how data saturation was considered, and how reflexivity was addressed during interpretation would strengthen methodological robustness.

**4. Theoretical and contextual clarifications**

**Gender Schema Theory**

The manuscript introduces GST appropriately, but its application could be more explicitly connected to the findings. For example, the claim that teachers' gender schemas shape pupil engagement (lines 387-389) is important and should be more deeply theorised, perhaps with references to how schema operate implicitly in classroom practices.

**Personal experiences of researchers**

The lead author's positionality (lines 124-131) mentions personal experiences with yoga/mindfulness but lacks elaboration. Were these experiences positive, challenging, or mixed? Greater context would clarify potential influences on the analytic lens.

**Teacher gender**: All participating teachers identified as female. The manuscript acknowledges this in Limitations but does not discuss its possible influence on pupils' participation or perception of yoga. This could be more directly addressed earlier in the discussion.

**Ethnic and cultural context**: Given the North-East England context and its socioeconomic profile (lines 144–149), a brief comment on ethnic or cultural diversity of the pupils (if available) would help assess how representative or generalisable the findings may be.

**5. Interpretation of findings**

**Assumption of gendered intent in teaching yoga**

The manuscript asserts that teachers may be using yoga to support social-emotional learning and that they may do so differently for boys and girls. This is a compelling claim, but it requires either clearer evidence from the data or greater support from existing literature.

**Mismatch between teacher and pupil perspectives**

The study finds that teachers perceive boys as less engaged, while pupils themselves (both genders) report enjoyment and participation. This is a critical insight that warrants further exploration, especially in light of potential adult gender schemas projecting onto pupil behaviour.

**6. Technical Criteria Compliance**

| **Criterion** | **Assessment** |
| --- | --- |
| Original Research | Yes |
| Previously Published | No |
| Analysis Standards | Needs more methodological transparency in coding and analysis presentation |
| Conclusions Supported | Partially, some claims need stronger evidence or clearer links to data |
| Language and Presentation | Clear and intelligible English throughout |
| Ethical Standards | Met, with approvals detailed |
| Data Availability | Compliant – available on request |
| Reporting Guidelines | SRQR followed, but coding details could be improved |

**7. Additional Suggestions**

**WHO definition of gender**

This appears late in the paper (line 166). It would be more appropriate to introduce the operational definition of gender earlier, ideally in the introduction or theoretical framework.

**Limitations Section**

Strong and honest. However, it could be further improved by acknowledging the absence of member checking or triangulation.

**8. Conclusion and Recommendation**

This manuscript offers valuable insights into a neglected area: gendered perceptions of yoga in early childhood education. However, several key elements need clarification or elaboration to meet the standards for qualitative research publications.

I recommend major revisions in accordance with the comments above.
