# Supplementary material for: Gendered perspectives of yoga in the Key Stage 1 classroom: Qualitative content analysis indicates contrasting views of teachers and pupils
Source: PLoS One. 2026 Feb 24;21(2):e0343622. doi: 10.1371/journal.pone.0343622 (PMC12931780; doi:10.1371/journal.pone.0343622)
Supplement: Attachment — Submitted filename: Response to Reviewers.docx [file pone.0343622.s005.docx]

The authors would like to thank all reviewers for their time and are grateful for their thoughtful, constructive comments. We have responded to all points made and believe that the paper is now much improved. N.B. Line numbers highlighted in yellow indicate lines in the ‘revised manuscript with track changes’ document. Line numbers without highlight indicate lines in the ‘revised manuscript’ document.

| Reviewer 1 | Responses |
| --- | --- |
| Introduction   - Because the research is UK based, it could be helpful to link to some UK stats on yoga participation: Cartwright, T., Mason, H., Porter, A., & Pilkington, K. (2020). Yoga practice in the UK: A cross-sectional survey of motivation, health benefits and behaviours. BMJ open, 10(1), e031848 | Introduction  Thank you for encouraging us provide this evidence of yoga participation in the UK. We have added this reference to the introduction section  Line 39  Line 38 |
| - Line 108-109 – when saying ‘much evidence’ can you link the reader to review level evidence rather than a single study? If no review level evidence is present, then is there more than one study you can refer to, otherwise use more cautious wording. | Thank you – we added three further references to back up this claim  Line 96  Line 95 |
| Methods   - It would have been helpful to have utilised a ‘critical friend’ from outside of the research team for objectivity – although I know that this can be difficult to access with limited researcher capability/capacity! Maybe something to consider for future qualitative research. | Methods  This is a very good point and is certainly something for us to consider in future. We have added a sentence to acknowledge this to the ‘Limitations’ section  Lines 583 – 586  Lines 539 – 542 |
| - Tables 1-2 – is it necessary to have the locations of the schools/teachers? If not necessary, I would remove to protect anonymity. You could classify the schools with number IDs and add ‘primary/infant’ etc. | Thank you, agreed. We have amended both tables accordingly |
| - Line 188-189 – Some additional detail on the assent processes would be helpful with the children being so young, how did you know they understood what was being asked of them? | This is an important point, thank you. We have added clarification of this to the ‘Pupil Qualitative Task’ section  Lines 220 – 222  Lines 213 – 215 |
| - Line 191 – How would you define contribution and ‘engaged’ in this setting? Did you have any tactics to involve those who may not have felt as comfortable engaging in the more traditional sense? | Again, this is an important point. We have added clarification of this to the Procedures section  Lines 186 – 189  Lines 179 – 181 |
| - Can you add some detail on justification of sample size for your study? | Information has been added to justify the sample size, thank you  Lines 197 – 198  Lines 190 – 191 |
| - Line 266 – not sure this reference is in the right place? | Thank you for spotting this. We have deleted this reference from the revised manuscript. |
| Findings   - Line 271-273 – Very hard to understand the meaning of this without knowing in-depth the method used – the figure helps slightly but I wonder if there is another way to describe this. - Quite interesting that the analysis of the pupils and teachers has been combined – can you detail why. Was it to avoid duplication or did the data complement each other? | Findings  Thank you, this was also highlighted by reviewer two. We have now included a table displaying key categories, codes, subcodes, and example quotes (Table 3).  The teachers’ and pupils’ data were analysed together to explore and illustrate how the findings from both populations are related. We have added this sentence to the Qualitative Content Analysis section to clarify this Lines 285 – 286  Lines 278 – 279 |
| - As you are discussing the findings with existing literature in the findings section, should you rename it ‘findings & discussion’? | Thank you. The title for this section has been amended to ‘Findings and Discussion’ |
| - Line 321-326 – Suggestion: because you are repeating a lot of the quotations to discuss, would there be a better way to format this? To avoid duplication could you present a quote and discuss, instead of presenting all quotes, then discussing them again? | Thank you, good point. I have removed two quotations to make this section less repetitive  Lines 337 and 340 |
| - Line 344 onwards – agree with this and would be interesting to discuss how yoga is marketed in this age group compared to other lifespan groups. For example, Cosmic kids is considered quite gender neutral and geared towards younger kids versus Studio You which is powered by This Girl Can. | This is useful, thank you. We have added a sentence to highlight this  Lines 372 – 376  Lines 363 – 367 |
| Conclusion   - This is written quite differently from typical journal articles and seems slightly repetitive with duplication of what we have just read including the references, rather than summarising the main takeaways. Is there a way to condense this section and make it slightly more punchy – the ‘so what?’ of the research needs to come out a bit more. | Conclusion  Thank you, we have re-written this section to make our take-away findings clearer and to minimise repetition |
| Limitations   - You could perhaps explore the heterogeneity of yoga and the difficulties of research because of this. | Limitations  Thank you, this is an important point to add. We have highlighted this issue in the limitations section  Lines 570 – 574  Lines 526 – 530 |

| Reviewer 2 | Responses |
| --- | --- |
| Title and framing   - Title Revision Required: The current title foregrounds the teacher’s view ("He saw it a bit like a dance...") and does not adequately reflect the dual focus of the study on both teachers and pupils. It risks misrepresenting the scope, which includes children’s perspectives. A more neutral and inclusive title is advised, ideally reflecting the mismatch between teacher and pupil perspectives. | Title and framing  This is a valid point, thank you. We have amended the title accordingly |
| Clarity and Transparency of Methods   - Missing content analysis table: The inclusion of a table summarising QCA categories and subcategories is essential. While a graphical coding frame (Figure 1) is referenced and supplementary materials are noted, the main manuscript would greatly benefit from a table displaying key categories, codes, subcodes, and example quotes, as per best practices in qualitative research reporting - Interview protocol: Although semi-structured interviews are described, the article would be enhanced by including example interview questions, particularly those that elicited gender-related insights. - Analytic elaboration needed: The explanation of the QCA process (p. 11,12) could be expanded. More detail on how consensus was reached, how data saturation was considered, and how reflexivity was addressed during interpretation would strengthen methodological robustness. | Clarity and Transparency of Methods  Thank you, this is something also highlighted by reviewer one. We have now included a table displaying the main category, two subcategories, and seven codes, with example quotes (Table 3).  Thank you. As described in the ‘Teacher Interviews’ section (lines 252 – 254, lines 245 – 247) the semi-structured interview was not initially designed or intended to explore gendered attitudes of yoga, but as the teachers made spontaneous mention of this issue, the interviewer responded to explore this. For clarity, we have included an example follow-up question  Lines 254 – 259  Lines 247 – 252  Thank you. We have added further details to this section as required Lines 271 – 278  Lines 264 – 271 |
| Theoretical and contextual clarifications   - Gender Schema Theory: The manuscript introduces GST appropriately, but its application could be more explicitly connected to the findings. For example, the claim that teachers' gender schemas shape pupil engagement (lines 387-389) is important and should be more deeply theorised, perhaps with references to how schema operate implicitly in classroom practices. - Personal experiences of researchers: The lead author's positionality (lines 124-131) mentions personal experiences with yoga / mindfulness but lacks elaboration. Were these experiences positive, challenging, or mixed? Greater context would clarify potential influences on the analytic lens. - Teacher gender: All participating teachers identified as female. The manuscript acknowledges this in Limitations but does not discuss its possible influence on pupils' participation or perception of yoga. This could be more directly addressed earlier in the discussion. - Ethnic and cultural context: Given the North-East England context and its socioeconomic profile (lines 144–149), a brief comment on ethnic or cultural diversity of the pupils (if available) would help assess how representative or generalisable the findings may be. | Theoretical & contextual clarifications  This is an important point, thank you for encouraging us to expand upon this. In responding to all reviewer comments, we now have more information relating to this throughout the manuscript  Lines 418 – 428, 438 – 443, 489 – 495  Lines 409 – 417, 427 – 432, 478 – 484  Thank you. Additional details have been added to the positionality section to cover this  Lines 117 – 119  Lines 116 – 118  Thank you. We have expanded the final paragraphs in the Findings and Discussion section to highlight this  Lines 492 – 495  Lines 481 – 484  Thank you. We have added these details to Table 1 and lines 153 – 156, lines 152 – 155 |
| Interpretation of findings   - Assumption of gendered intent in teaching yoga: The manuscript asserts that teachers may be using yoga to support social-emotional learning and that they may do so differently for boys and girls. This is a compelling claim, but it requires either clearer evidence from the data or greater support from existing literature. - Mismatch between teacher and pupil perspectives: The study finds that teachers perceive boys as less engaged, while pupils themselves (both genders) report enjoyment and participation. This is a critical insight that warrants further exploration, especially in light of potential adult gender schemas projecting onto pupil behaviour | Interpretation of findings  Thank you. We have added further references to demonstrate that yoga is sometimes used to support pupils’ social-emotional learning in school, but have ended the paragraph by saying that further research is required to clarify this issue  Lines 418 – 428  Lines 409 – 417  We have expanded this paragraph to discuss this finding in greater depth  Lines 438 – 443  Lines 427 – 432 |
| Additional Suggestions   - WHO definition of gender: This appears late in the paper (line 166). It would be more appropriate to introduce the operational definition of gender earlier, ideally in the introduction or theoretical framework | Additional Suggestions  Good point. We have moved this information so that it is presented earlier in the paper as you advise  Lines 79 – 83  Lines 78 – 82 |
| Limitations Section   - Could be further improved by acknowledging the absence of member checking or triangulation. | Limitations Section  Thank you, these are important points. We have included sentences to highlight this to the limitations section  Lines 582 and 584  Lines 538 and 540 |
| Reviewer 3 | Responses |
| - I have some second thoughts regarding the conclusion that "any potential gendering of yoga is not fixed and is not considered accurate or appropriate by the majority of these five- and six-year-old pupils" (Line 341-342). It is not obvious that the age of the pupils who express a non-stereotypical gendered view of yoga is around 6 years, as the Gender Schema Theory suggests on Line 337-339 (Table 2, page 8 offers a participant-age spectrum from infant-school up to first-school age). - 9 out of 23 pupils say that yoga is not for any particular gender or subset of the population, and 10 out of 23 pupils say that yoga is ‘for’ their gender. That leads me to question that any potential gendering of yoga is not fixed and is not considered accurate or appropriate by the majority of these five- and six-year-old pupils (Line 341-342). - As the data analysis is based only on the verbally expressed gender-based stereotypical beliefs by the pupils, it seems possible that more (non) gender stereotypical beliefs exist that were not communicated verbally by the pupils and are not taken into consideration. I am reluctant to conclude that the majority shares this belief. | Thank you for pointing out that this is unclear. As shown in Table 2, the pupil participants attended an infant school (with pupils up to 7 years of age) and a first school (with pupils up to 9 years of age) however it is clarified (in line 152, line 151) that all pupil participants were five or six years of age. I have updated the asterisked text beneath Table 2 to clarify this  Lines 169 – 170  Lines 162 – 163  Thank you, we have now worded this assertion more carefully to better reflect the data  Lines 361 – 362  Lines 353 – 354  Thank you. We have added details to the limitations section in an attempt to address this issue  Lines 576 – 577 and 582  Lines 532 – 533 and 538 |
| - The data underlying the results presented in the study are available from the lead author via email. I am wondering whether the video recordings and transcriptions can be found in an open source - Will the pupil qualitative task materials be utilized somehow (this study's findings derive only from the verbal statements of the pupils, if I understood correctly)?      - Regarding the pupil qualitative task materials, I am wondering whether a case of a pupil arose that s/he chose a figure non-identical to their sex/gender, but their verbal statements came from their gendered views (Line 208-209). | As per safeguarding and data protection regulations, it is not appropriate to share video recordings of the young pupils’ task participation. Also, as the study covers potentially sensitive topics (gendering / gender stereotypes) the authors would prefer not to make all data publicly available without restriction. It will be possible for interested parties to request access to the anonymised participant transcriptions by emailing a member of the University’s library services team who has responsibility for handling data access requests  Analysis of the children’s artwork is beyond the scope of this paper, so we do not include any analysis of the drawings created by the children in this manuscript. Our rationale for this is provided in lines 240 – 244, lines 233 – 237  This is an interesting point, thank you. We have now included information relating to this  Lines 208 – 210  Lines 201 – 203 |
| - Gender Schema Theory is the primary theoretical framework employed. The author also mentions alternative theories (Line 118-120). It would be interesting to include the names of alternative theories in the text, not just the given citations. | Thank you for making this point, we have updated this section accordingly  Line 106  Line 105 |
| - It would be great to have an interpretation about the female and non-male teacher participation (Line 162-163). - Also, a comment to be included about the lack of primary school participants (Table 2, page 8) - Lastly, adding an explanation of the Ofsted rating as a note (Table 2, page 8) would be helpful. | Thank you. This was also highlighted by reviewer two. We have added some information to address this  Lines 492 – 495  Lines 481 – 484  Thank you, we have added information to address this  Lines 373 – 379  Lines 364 – 370  Good point, thank you. We have added an explanation beneath Table 2  Lines 172 – 173  Lines 165 – 166 |
| - It is truly valuable that the teachers in this study work at schools with disadvantaged youth. I would like to read a relevant comment on gendered views of yoga and disadvantaged youth (Line 148-149). | Thank you, we have added a final paragraph to the Limitations section to highlight this  Lines 588 – 592  Lines 544 – 548 |
| - It was a little unclear how the video recordings help with the contribution assurance (Line 203-205). | Thank you. We have expanded this explanation  Lines 202 – 203  Lines 195 – 196 |
| - Under the Teacher’s views on yoga and gender section, two opposing perspectives appear: yoga offers an individual space, versus yoga is seen as a team effort. Could these perspectives be interpreted from the GST perspective? | This is an interesting point and could be evaluated in more depth. However, to avoid losing focus, we have opted not to add any further discussion of this finding |
